# Anti-viral drug discovery against monkeypox and smallpox infection by natural curcumin derivatives: A Computational drug design approach

**DOI:** 10.3389/fcimb.2023.1157627

**Published:** 2023-03-22

**Authors:** Shopnil Akash, Arafat Hossain, Md. Sarowar Hossain, Md. Mominur Rahman, Mohammad Z. Ahmed, Nemat Ali, Martin Valis, Kamil Kuca, Rohit Sharma

**Affiliations:** ^1^ Department of Pharmacy, Faculty of Allied Health Science, Daffodil International University, Dhaka, Bangladesh; ^2^ Department of Biochemistry and Molecular Biology, Bangabandhu Sheikh Mujibur Rahman Science and Technology University, Gopalganj, Bangladesh; ^3^ Department of Pharmacognosy, College of Pharmacy, King Saud University, Riyadh, Saudi Arabia; ^4^ Department of Pharmacology and Toxicology, College of Pharmacy, King Saud University, Riyadh, Saudi Arabia; ^5^ Department of Neurology, Medical Faculty, Charles University and University Hospital in Hradec Králové, Hradec Králové, Czechia; ^6^ Department of Chemistry, Faculty of Science, University of Hradec Králové, Hradec Králové, Czechia; ^7^ Biomedical Research Center, University Hospital Hradec Kralove, Hradec Kralove, Czechia; ^8^ Department of Rasa Shastra and Bhaishajya Kalpana, Faculty of Ayurveda, Institute of Medical Sciences, Banaras Hindu University, Varanasi, Uttar Pradesh, India

**Keywords:** curcumin, monkeypox, smallpox virus, molecular docking, DFT, admet, molecular dynamic simulation

## Abstract

**Background:**

In the last couple of years, viral infections have been leading the globe, considered one of the most widespread and extremely damaging health problems and one of the leading causes of mortality in the modern period. Although several viral infections are discovered, such as SARS CoV-2, Langya Henipavirus, there have only been a limited number of discoveries of possible antiviral drug, and vaccine that have even received authorization for the protection of human health. Recently, another virial infection is infecting worldwide (Monkeypox, and Smallpox), which concerns pharmacists, biochemists, doctors, and healthcare providers about another epidemic. Also, currently no specific treatment is available against Monkeypox. This research gap encouraged us to develop a new molecule to fight against monkeypox and smallpox disease. So, firstly, fifty different curcumin derivatives were collected from natural sources, which are available in the PubChem database, to determine antiviral capabilities against Monkeypox and Smallpox.

**Material and method:**

Preliminarily, the molecular docking experiment of fifty different curcumin derivatives were conducted, and the majority of the substances produced the expected binding affinities. Then, twelve curcumin derivatives were picked up for further analysis based on the maximum docking score. After that, the density functional theory (DFT) was used to determine chemical characterizations such as the highest occupied molecular orbital (HOMO), lowest unoccupied molecular orbital (LUMO), softness, and hardness, etc.

**Results:**

The mentioned derivatives demonstrated docking scores greater than 6.80 kcal/mol, and the most significant binding affinity was at -8.90 kcal/mol, even though 12 molecules had higher binding scores (-8.00 kcal/mol to -8.9 kcal/mol), and better than the standard medications. The molecular dynamic simulation is described by root mean square deviation (RMSD) and root-mean-square fluctuation (RMSF), demonstrating that all the compounds might be stable in the physiological system.

**Conclusion:**

In conclusion, each derivative of curcumin has outstanding absorption, distribution, metabolism, excretion, and toxicity (ADMET) characteristics. Hence, we recommended the aforementioned curcumin derivatives as potential antiviral agents for the treatment of Monkeypox and Smallpox virus, and more in vivo investigations are warranted to substantiate our findings.

## 1 Introduction

Viruses have been considered obligatory microscopic biological infections that are incredibly tiny and pathogenic (Greenwood et al., 2012). They are very complex nonliving substances or basic biological microorganisms that may act as live-in host cells and act as a particle outside the host cell (Villarreal, 2004). They are obligatory intracellular parasitic since they do not possess the metabolic enzymes or the infrastructure necessary to produce proteins (Vlachakis et al., 2013). The viral genome is formed of a single form of nucleic acid, either deoxyribonucleic acid (DNA) or ribonucleic acid (RNA), as well as a capsid (Lucas and Knipe, 2010). The protein coat is occasionally surrounded by an enclosure that is made up of lipids, proteins, and carbohydrates (Parvez, 2020). Viruses can infect every living thing, such as animals, plants, bacteria, and archaea6, and can only multiply and execute through their physiological activities in a host cell.

In the last couple of years, viral disease has developed very rapidly and infected millions of people around the globe, such as SARS CoV-2 (Khattak et al., 2022; Miller et al., 2023), Monkeypox virus (Mahase, 2022), Ebola virus (Malvy et al., 2019), HIV (Zhou and Saksena, 2013), Smallpox (Smith and McFadden, 2002), Hantavirus (Vaheri et al., 2013), Influenza (Dunn and Miller, 2014), Langya Henipavirus (Akash et al., 2022b), and Dengue (Pawitan, 2011). This viral infection is sometimes converted into global pandemics such as the SARS-CoV-2 pandemic and the Ebola pandemic (Islam et al., 2022), which directly impact world economics and the health sector (Kumer et al., 2022b). Several studies and findings have reported that millions of people die annually due to viral infection.

The *Orthopoxvirus* genus of the *Poxviridae* family includes the enveloped double-stranded DNA virus known as the monkeypox virus. Monkeypox is a viral zoonosis that is clinically less severe than Smallpox. It has symptoms comparable to those of Smallpox (Di Giulio and Eckburg, 2004; Rizk et al., 2022). The Monkeypox virus is susceptible to several animal species. A 9-month-old boy in the Democratic Republic of the Congo, where Smallpox had been eradicated in 1968, was the first person to be diagnosed with human Monkeypox in 1970 (Breman et al., 1980). Contacting infected animals’ blood, body fluids, skin, or mucosal lesions can result in animal-to-human (zoonotic) transmission. Since it affects the rest of the world, in addition to nations in west and central Africa, Monkeypox is a disease of worldwide public health concern now (Di Giulio and Eckburg, 2004; McCollum and Damon, 2014).

The World Health Organization declared Monkeypox an “evolving threat of moderate public health concern” on June 23, 2022, after more than 3000 Monkeypox virus infections were detected in more than 50 nations (Thornhill et al., 2022). There isn’t a specific medication for the Monkeypox virus infection right now. However, a number of antiviral drugs used to treat Smallpox and other ailments could be beneficial for those with Monkeypox infection. According to the Centers for Disease Control and Prevention (CDC), supportive care is often sufficient for people with a Monkeypox virus infection because no particular medicines are available (Rizk et al., 2022). The computational technique is used to develop a safe drug against Monkeypox and Smallpox virus infection. Because developing any medication takes more than 10-12 years, huge cost, time, and resources, but this new computational technology creates a new era in which effective drugs and new biological substances can be developed within a concise period of time, reducing both time and costs (Rahman et al., 2012; Tropsha and Bajorath, 2016; Stanzione et al., 2021). For more than three decades, the discovery of clinically significant compounds has been greatly helped by computer-aided drug discovery and design techniques (Marhöfer et al., 2011; Hung and Chen, 2014). Although, computer-aided drug discovery and design has a lot of advantages and reduce the time, cost and resources to develop a potential drug, but it has some its own limitations.

One of the most complicated issues has to be solved in drug development is considered about target flexibility. The use of a toxicity prediction model is helpful in determining whether or not a medication candidate is harmful to organs such as the liver, kidneys, heart, and lungs. Besides, the accuracy of prediction models is hindered by a lack of trustworthy experimental data and factors relevant to ADME and toxicity. It is also noteworthy to know that only forty percent of medicine candidates are being tested in clinical trials by the forecast computer model (Singh, 2014; Hassan Baig et al., 2016).

Curcumin and its derivatives are thought to have carried out a study against Monkeypox and Smallpox infection. According to research, curcumin, the primary biologically active component of turmeric (*Curcuma longa L*.), functions as a potent anti-inflammatory, antioxidant, antibacterial, antifungal, and antiviral activity (Rathore et al., 2020). The chemical formula C_21_H_20_O_6_ may represent it, and its molecular mass is determined to be 368.38 g/mol. This lipophilic polyphenol is principally abundant in the rhizomes of turmeric (*Curcuma longa L)*, which is a member of the ginger family (Zingiberaceae) and is indigenous to the tropical regions of South Asia. It contains a yellow-to-orange hue. Turmeric powder is an eastern flavor that is typically derived from this plant. Curcumin, along with natural ingredients and other coumarins, is one of the most prominent bioactive substances recognized in turmeric powder (Basnet and Skalko-Basnet, 2011; Kotha and Luthria, 2019; Adamczak et al., 2020). Due to its antimicrobials, antioxidative, anti-inflammatory, and anti-cancerous properties, curcumin has long been anticipated to be a therapeutic or preventive agent for many human diseases (Tønnesen and Karlsen, 1985; Hsu and Cheng, 2007; Hatcher et al., 2008; Lestari and Indrayanto, 2014).

## 2 Literature review of curcumin

### 2.1 MPXV: Genome and physiological characteristics

**Figure 1:**
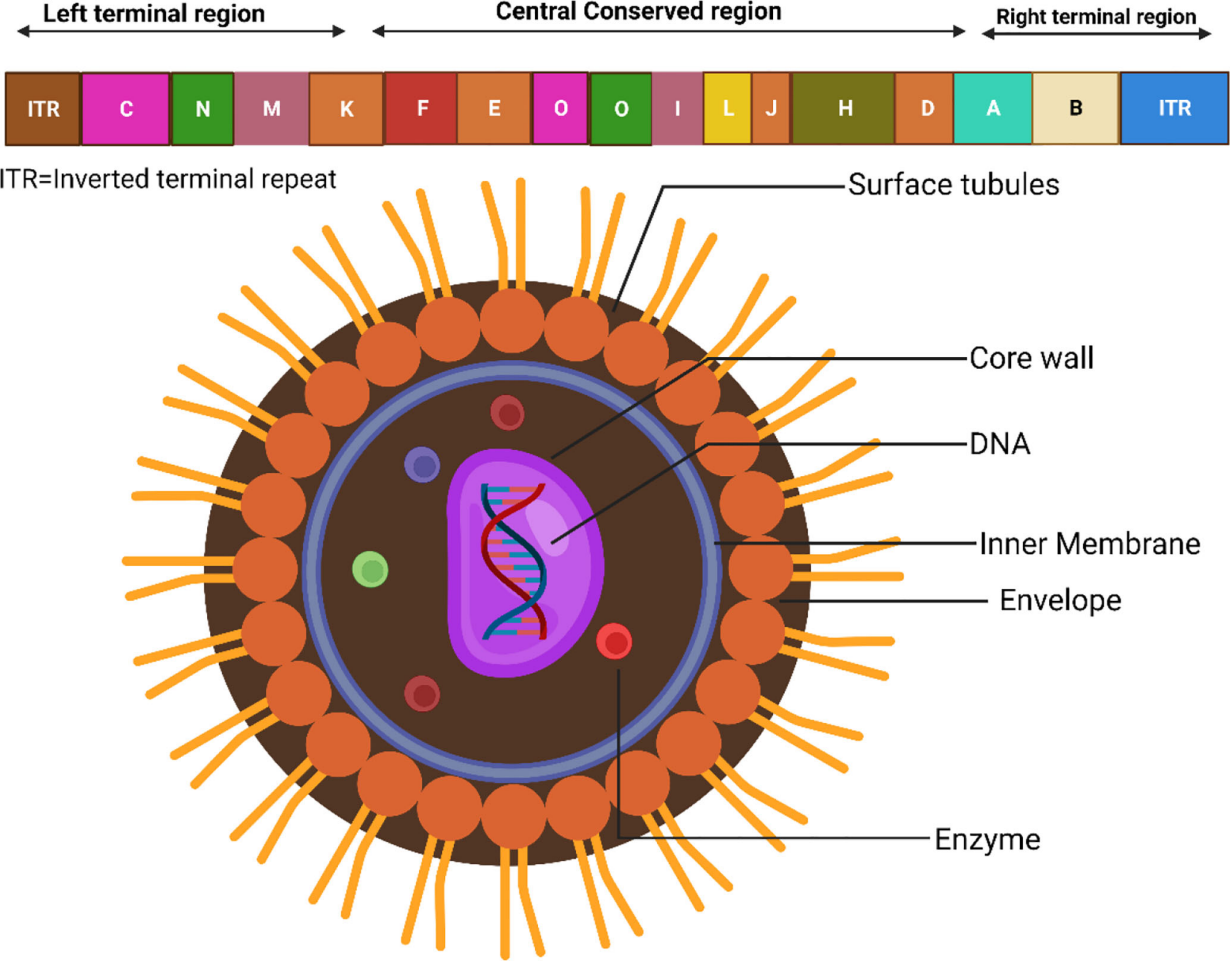
Physiological structures of MPXV.

Since its first appearance, the Monkeypox virus (MPXV) has been found throughout West and Central Africa. Recently, occasional cases of MPXV infections have been reported in a number of nations. According to recent findings, the MPXV-2022 strains are members of the same family as the MPXV strain discovered in 2018. In comparison to the MPXV strain that was found in 2018, the MPXV-2022 strains have been shown to include a total of 46 additional consensus substitutions, comprising 24 variants that are nonsynonymous (Wang et al., 2022). Besides, the MPXV genome consists of about 197,000 base pairs and has hairpin termini in addition to more than 190 open reading frames that do not overlap (ORFs) (Shchelkunov et al., 2001). The highly conserved segment in the middle of the genome that codes for proteins is surrounded on both sides by flexible endpoints that include inverted terminal repeats. At least ninety open reading frames (ORFs) are documented to be necessary for the proliferation and development of poxviruses. Many other ORFs considered non-essential have a role in the abnormalities in host tropism, immunomodulation, and pathogenicity caused by poxviruses (Seet et al., 2003). The form of MPXV virions may be described as either barrel or oval, and their sizes range from around 280 to 220 nanometers in general (Erez et al., 2019; Kmiec and Kirchhoff, 2022). The physiological features and structures of MPXV are displayed in **Figure 1** (Miller, 2013).

### 2.2 Effect of curcumin on viral infections

Curcumin has been shown to interfere with the progress of viral infection *via* various pathways, explicitly targeting the viral genome, reducing protein assembly and cell proliferation, and preventing the virus from entering the host cells and replicating (Basu et al., 2013; Wang et al., 2020). An updated research investigation reported that curcumin might be suppressed the respiratory syndrome virus (RSV) by preventing the virus’ interaction with hosting cells (Yang et al., 2017). According to new findings, curcumin seems to hinder the attachment of the porcine reproductive and respiratory syndrome virus (PRRSV). This may be accomplished by affecting the flexibility of viral envelopes. In addition, curcumin can prevent viral infection by blocking PRRSV-mediated cell fusion (Du et al., 2017). Another study reported that curcumin and its derivatives had been shown to have a high binding affinity to hemagglutinin (HA), the primary capsid glycoprotein of the influenza virus that is responsible for viral attachment, and disrupts the integrity of the membrane structure to inhibit IAV entrance. This stops the virus from adhering to host cells and prevents the virus from entering the cell (Liu and Ying, 2020).

### 2.3 Effects of curcumin on the SARS CoV-2

The primary antiviral function of curcumin against SARS-CoV-2 is its capacity to block the folding of viral spike protein to the ACE2 receptors, which is the first stage in the development of infecting the host organism. The inflammatory response brought on by COVID-19 is a complicated and multi-step phenomenon. Patients with a severe illness are more likely to be influenced by a hyperinflammatory phenomenon known as a cytokine storm. This fact highlights the necessity for anti-inflammatory therapies to minimize the hyperactivation of the immune reaction that occurs when it causes the cytokine storm (Rattis et al., 2021). Two investigations were performed with COVID-19-infected patients, one of which focused on the anti-inflammatory effects of curcumin. The first investigation that the research team undertook looked at how nano curcumin affected the production of cytokines that promote inflammation. Patients diagnosed with COVID-19 had an extreme amount of mRNA generation and production of the cytokines IL-1β, IL-6, TNF-α, and IL-18; however, patients treated with nano curcumin exhibited a substantial decline in IL-6 and IL-1β levels (Valizadeh et al., 2020; Tahmasebi et al., 2021).

### 2.4 Curcumin function on dengue protease

Research and investigation reported that curcumin might inhibit plaque formation caused by dengue virus strains (DENV-1-4, IC50 of 9.37, 3.07, 2.09, and 4.83 M, correspondingly) that were tested in LLC-MK2 cells and exhibited only a moderate level of toxicity (CC50 of 59.42 M) (Gao et al., 2019). Even though the method of suppression was not investigated, previous research indicated that curcumin probably suppresses DENV-2 implicitly *via* its influence on cellular systems rather than immediately on viral activities. Besides, curcumin and its four analogs could suppress viral protease activity (IC50 values ranged from 36–66 M) when tested in an *in vitro* experiment. The multiplication of a DENV2 reporter plasmid mutant was only moderately suppressed by these substances, with the acyclic and cyclohexanone analogs of curcumin functioning substantially better than the natural curcuminoids (50% effective concentration (EC50) of 8.61 and 8.07 µM vs. 13.91 µM) (Balasubramanian et al., 2019). It appears that the actions of curcumin on cellular lipid metabolism were responsible for the virus-inhibiting properties of curcumin against DENV. Curcumin and its analogs could inhibit the enzymes acetyl-CoA carboxylase and fatty acid synthase and reduce the development of lipid droplets (LD). These mechanisms would ordinarily serve to make DENV acquisition more prevalent. In addition, therapy with curcumin culminated in the disarray of actin filaments and abnormalities in polymerization, which is another function that is inherently essential for DENV entrance and reproduction (Balasubramanian et al., 2019; Jennings and Parks, 2020).

### 2.5 Effects of curcumin on the influenza A virus

Curcumin is another potent inhibitor of the Influenza A virus (IAV), and it probably impacts the virus at various phases during its lifespan. When IAV is incubated with curcumin, the pathogenicity of the virus is diminished; this is presumably owing to the capacity of curcumin to compromise with the viral haemagglutinin function (Chen et al., 2010; Han et al., 2018). Curcumin significantly blocks NF-kB signaling, which is essential for the reproduction of the influenza A virus (Nimmerjahn et al., 2004). For instance, curcumin blocked various IAV-induced toll-like receptor (TLR) signaling pathways and enzymes, such as TLR2/4/7, MyD88, TRIF, and TRAF6, which are generally essential for effective virus assembly. Stimulating cells with agonists for TLR2/4, p38/JNK MAPK (Dai et al., 2018).

### 2.6 Effects of curcumin on Zika, and chikungunya virus

Several curcumin analogs that are effective against the enveloped viruses Zika (ZIKV), Chikungunya (CHIKV), and Vesicular Stomatitis (VSV), as well as the non-enveloped virus Coxsackie B3 (CVB3) (Adams et al., 2004). Curcumin could be effective in inhibiting when applied to cells both before and after being infected with Zika or chikungunya; however, it works against Zika solely during cell attachment or entrance and does not affect the final stages of the disease (Ardebili et al., 2021). Curcumin at concentrations of 5 M was demonstrated to be significantly efficient. This resulted in a drop in virus intensity of more than 0.5 log10 despite causing negative impacts. Additionally, the IC50 values for curcumin against Zika and Chikungunya were significantly 1.9 M and 3.89 M (Mounce et al., 2017). In addition, they encountered that curcumin stopped the chikungunya and vesicular stomatitis virus from entering cells or attaching to them (von Rhein et al., 2016).

### 2.7 Effects of curcumin on herpes simplex virus

Components of curcumin that include -unsaturated ketone groups make the HCV membranes less flexible. This, in turn, prevents the virus from attaching to cells and fusing with them. Therefore, curcumin prevents the entry of all HCV genotypes into cells that have been evaluated in a dose-dependent manner, with a half-maximal inhibition zone (IC50) of about 8.46 1.27 M (Colpitts et al., 2014; Qin et al., 2014). Curcumin’s ability to block the PI3K-AKT and Akt-SREBP-1 pathways and induce heme oxygenase is responsible for its antiviral effects (Calland et al., 2015; Wahyuni et al., 2018).

### 2.8 Curcumin as HIV protease inhibitor

Curcumin has been the subject of a significant number of research, all of which have shown to be an effective inhibitor of HIV protease. Curcumin meanly inhibit HIV-1 proteases (IC50 = 100 M) and HIV-2 proteases (IC50 = 250 M), which may be responsible for its anti-HIV activities (Prasad and Tyagi, 2015). By engaging with the catalytic center of isolated HIV-1 integrase, curcumin was capable of inhibiting HIV-1 integrase with an IC50 value of 40 microM. Additional research demonstrated that the anti-integrase action of curcumin was connected to the intramolecular arrangement of two phenyl rings, which brought the hydroxyl groups into direct connection with one another (Prasad and Tyagi, 2015). Not only does curcumin control the infectious potential of HIV, but it also boosts the potency of medications used to treat HIV and AIDS. In another research, curcumin was demonstrated to increase the systemic exposure of saquinavir in rats, but it did not affect the intravenous pharmacokinetics of saquinavir (Kim et al., 2013). In the appearance of the curcumin-loaded microemulsion, the oral administration of saquinavir led to a rise in both the AUC and Cmax by a factor of 3.8 and 2.7, significantly (Prasad and Tyagi, 2015).

**Figure 2:**
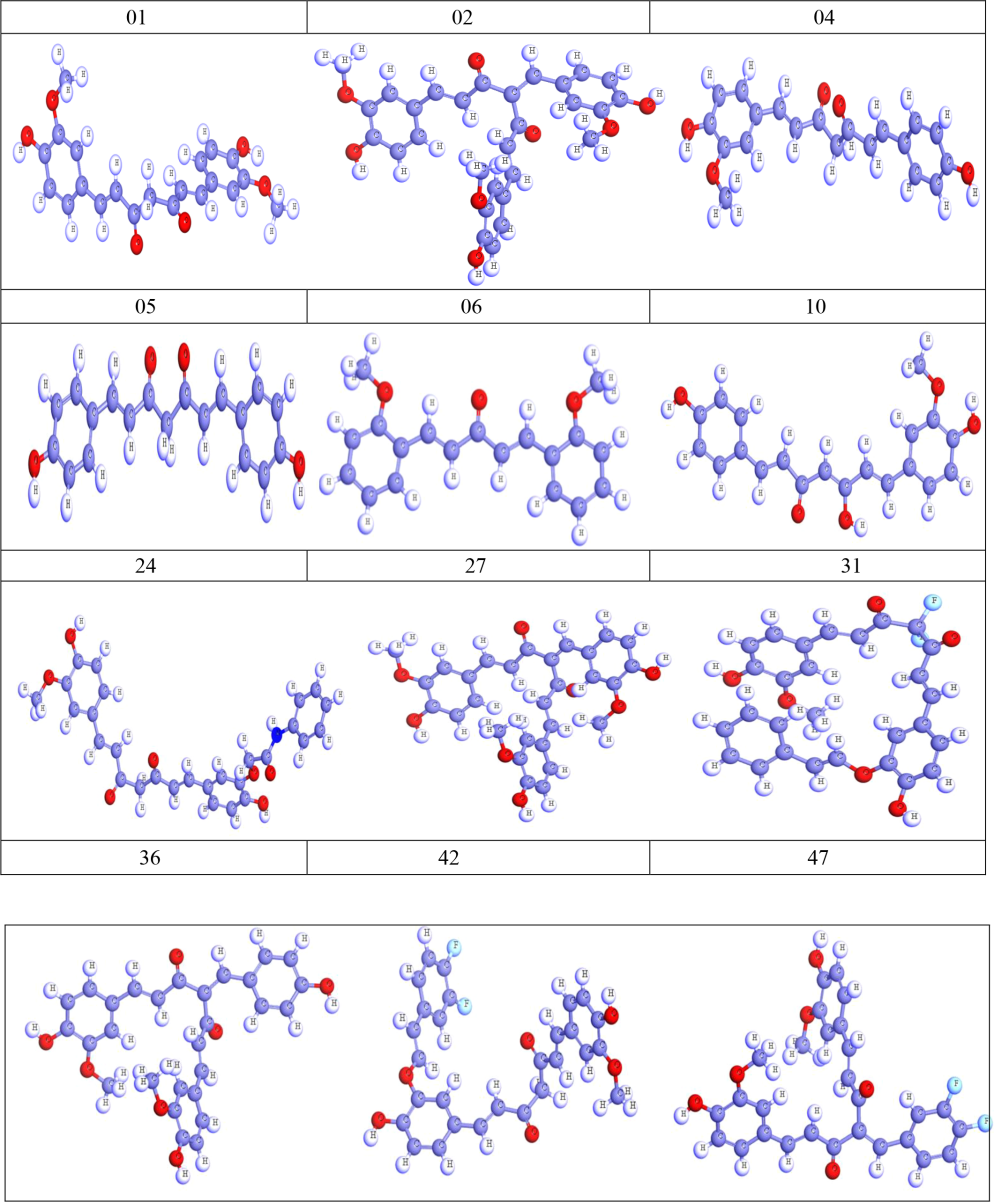
Optimized structure of curcumin derivatives.

**Figure 3:**
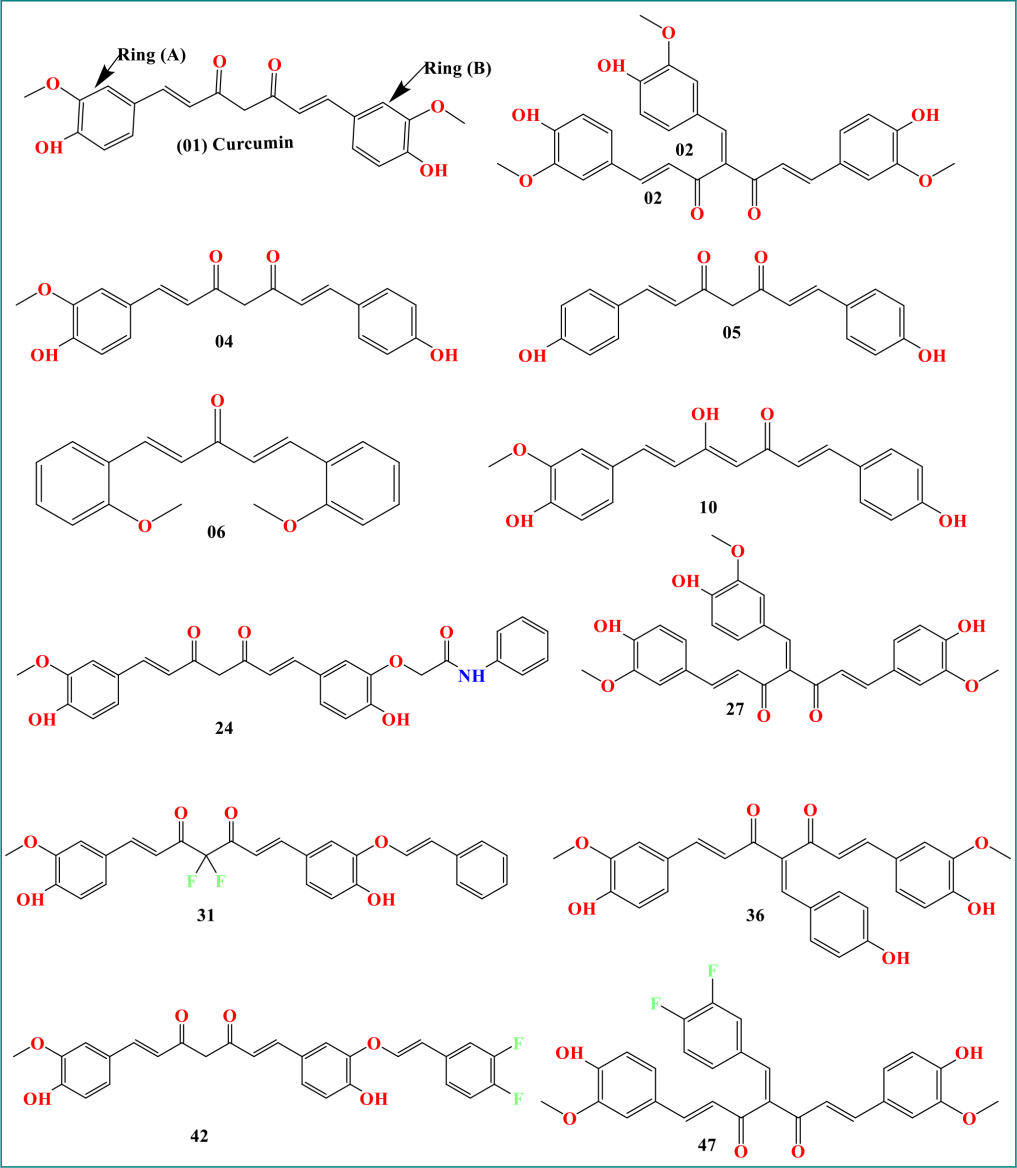
Chemical structure of curcumin and its derivatives.

**Figure 4:**
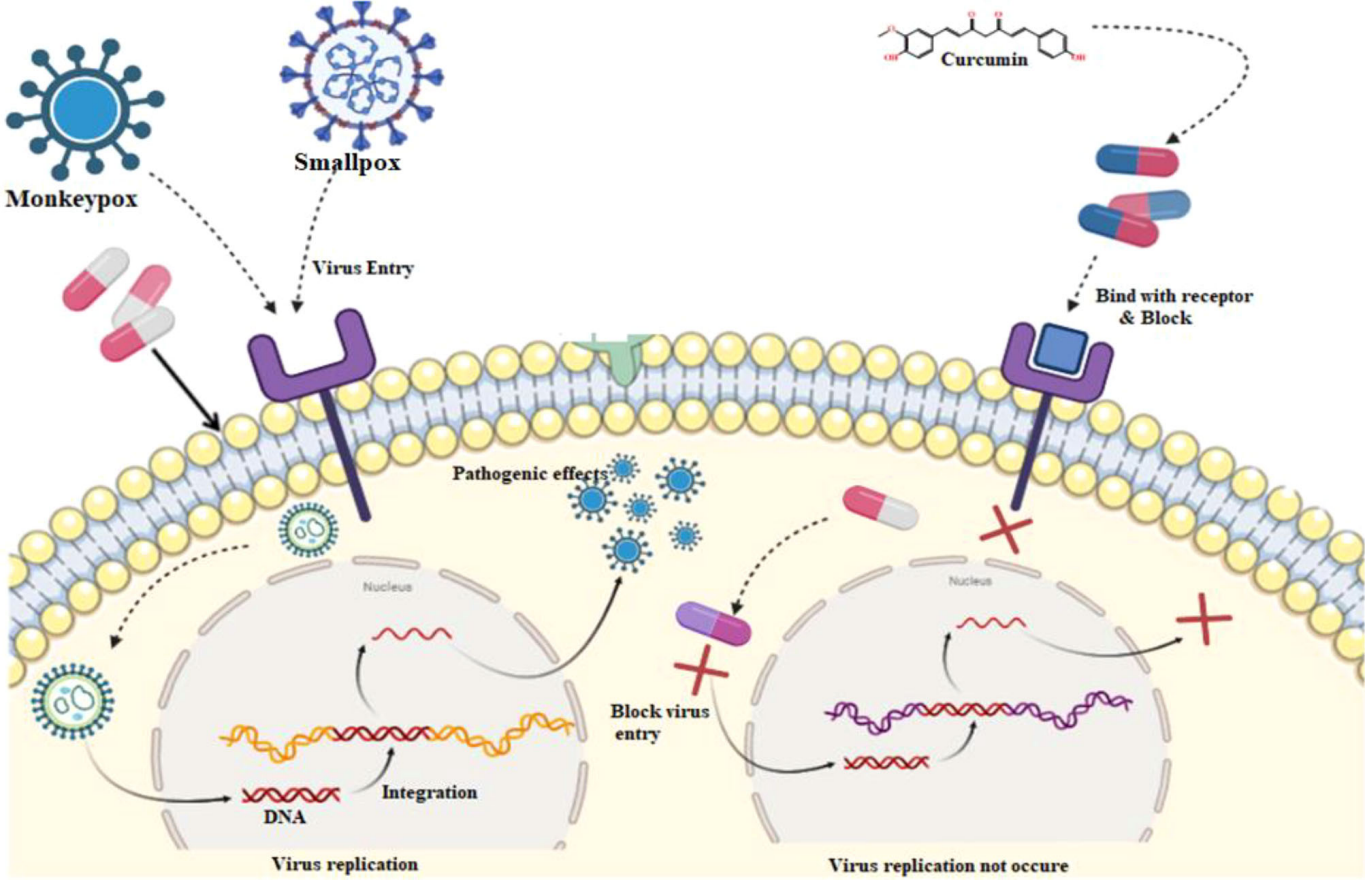
Probable mechanism action of curcumin against the virus.

As curcumin is already established as potential effectiveness against the different viruses in clinical and laboratory trials (**Table 1**), so, we believe that the curcumin derivatives might have capabilities to inhibit viral entry/gene expression. Thus, we have selected more than 50 curcumin derivatives available in natural sources to find an effective medication against smallpox and Monkeypox virus treatment. At the beginning of the studies, these 50 derivatives were conducted molecular docking against the Monkeypox virus and selected best 12 compounds according to maximum binding energy for further investigation (**Figures 2**, **3** displayed selected the most potent 12 curcumin derivatives from 50 natural curcumin analogs, **Figure 4** is illustration for antiviral mechanism of curcumin).

**Table 1:** Experimental and clinical data of antiviral activities against viral strains reported in different research investigations (throughout 2018-2022).

No	Virus species	Antiviral Activity	References
01	SARS CoV-2	Inhibiting the Endosomal acidification	(Rattis et al., 2021)
02	Dengue virus	Entry inhibitor	(Balasubramanian et al., 2019)
03	Influenza A virus	Replication inhibitor	(Dai et al., 2018)
04	Herpes simplex virus	Gene expression inhibition	(van de Sand et al., 2021)
05	Human immunodeficiency virus	Viral protein degradation/Protease inhibitor	(Butnariu et al., 2022)
06	Zika virus	Entry inhibitor	(Pacho et al., 2021)

The phases of the viral life cycle are complex and occur through different processes. These processes consist of the attachment of the virion to its cell surface receptor, the subsequent entrance of the virion, the phase of viral genome replication and transcription, the process of translation of the viral genome, the congregation of the virion, and ultimately the release of the virion. As a result of curcumin’s ability to suppress the function of viral envelope proteins, viral attachments and entrance are both blocked.

Furthermore, curcumin has been shown to affect specific signaling pathways, inflammatory processes, and translation and transcription, which ultimately results in a blockage of viral replication. Then, curcumin competitively inhibits the synthesis of viral DNA within the host cell. As a result, the virus cannot multiply and is unable to survive within the host cell (Hussain et al., 2022).

### 2.9 Transmission and clinical manifestation

**Figure 5:**
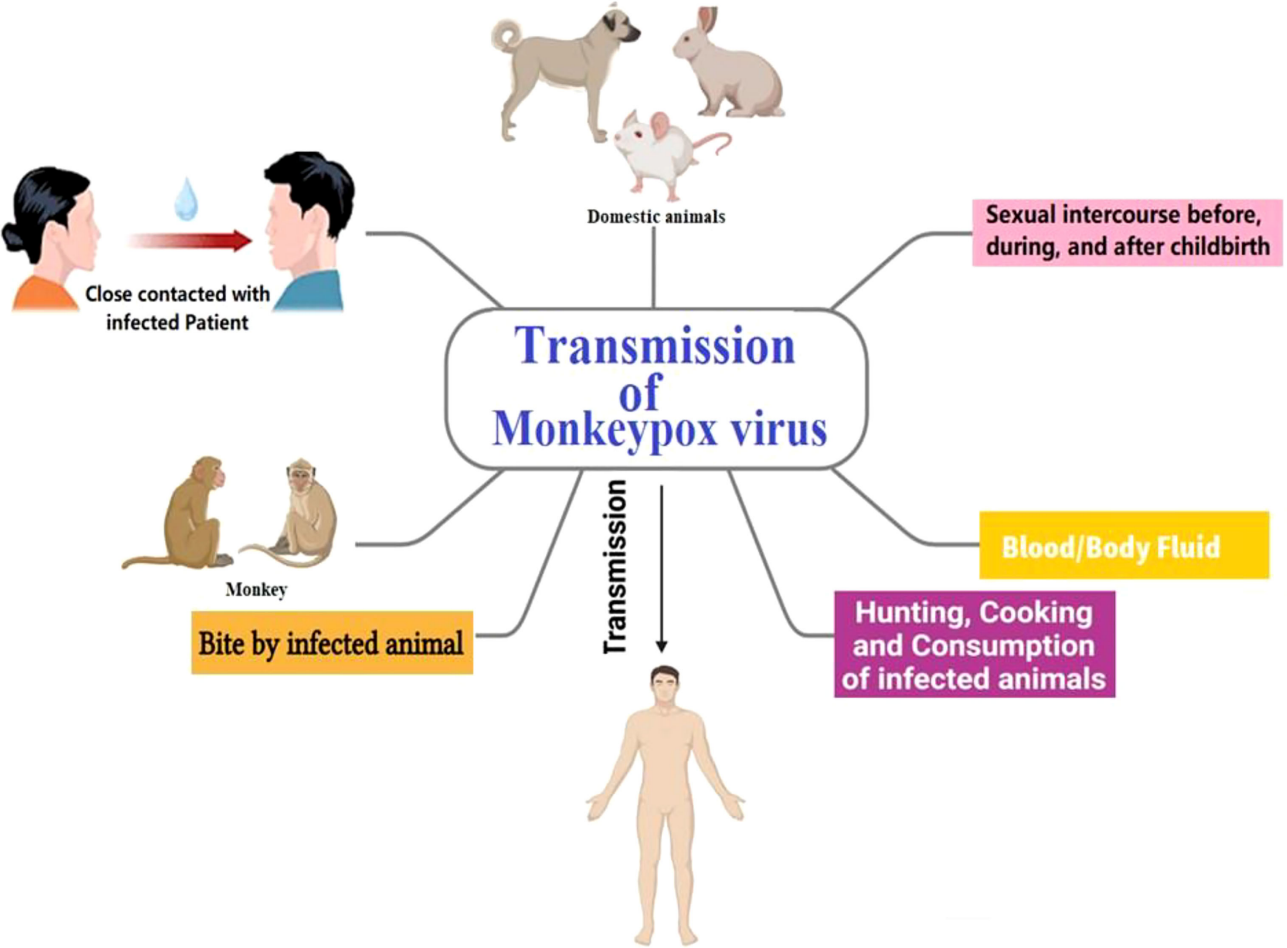
Monkeypox transmission pathway.

The natural reservoir of MPX has not been identified; however, rats are a leading possibility. Consumption of uncooked or improperly prepared meat or other animal products from infected animals is a potential risk factor. People who reside in or near forested areas may also be at risk of low-level or indirect exposure to diseased animals. Though transmission of MPX is infrequent, it may occur *via* direct or indirect contact with infected bodily fluids (Guarner et al., 2022). The MPX may be transmitted *via* the placenta and spread through sexual intercourse before, during, and after childbirth (Di Gennaro et al., 2022). Rash, fever, chills, headache, adenopathy, and myalgia have been the ailments and indicators of MPX disease (Sale et al., 2006). Occasionally, in the first stages of MPX disease, the rash has present primarily in the genitalia and perineal regions. The incubation period for Monkeypox may be from around 5 to 21 days, although it is most often between 6 and 13 days. After 14–21 days, in most cases, patients recover completely from the disease on their immunity (Kumar et al., 2022; Pal et al., 2017). In **Figure 5**, the transmission pathway of MPX is graphically displayed.

## 3 Computational method

### 3.1 Preparation of Ligand and geometry optimization

Curcumin is a natural flavonoid polyphenol class compound, which is the primary and bioactive component of turmeric. Around 8000 different flavonoid compounds have been discovered, making flavonoids the most abundant family of phenolic chemicals. In addition to this, they are considered nutritional supplements that improve health and guard against infection (Zeghbib et al., 2022). Numerous studies demonstrate that curcumin reflects a variety of biological actions, suggesting that they may have preventive benefits against a broad range of diseases (**Table 1**). In this investigation, more than 50 compounds were taken from the PubChem database (https://pubchem.ncbi.nlm.nih.gov/) (50 derivatives displayed in **Supplementary Table 1**), and the canonical SMILES of the exploration molecules were also acquired from the PubChem database. The 2D structures of the molecules were obtained by importing the canonical SMILES into the ChemBioOffice program. The Molecular optimization of 3D structure of molecules were carried out with the assistance of a method known as density functional theory (DFT) then optimized geometrically, and energy was minimized by employing B3LYP and the functional unit DFT procedure of DMoL3 code from material studio 08 (Delley, 1995; Delley, 2010; Ramos, 2020). The HOMO-LUMO expression was then calculated using the optimized structures, and the optimized molecules were saved in PDB format for further work. The optimized chemical structures of curcumin derivatives are given in **Figure 2**.

### 3.2 ADMET profile prediction, Lipinski rule, and drug-likeness

The advancement of computer technology has enabled scientists to create innovative drug targets, which has cut down on the number of experiments that need to be conducted while simultaneously raising the success rate; in order to facilitate preliminary evaluation during drug discovery and development, ADMET pharmacokinetic features and drug-likeness play a major role. The ADMET (Absorption, Distribution, Metabolism, Excretion, and Toxicity) characteristics might be obtained using the in silico investigation (Kumer et al., 2021; Hassan et al., 2022). In this current study, the pkCSM(https://biosig.lab.uq.edu.au/pkcsm/) and SwissADME (http://www.swissadme.ch/index.php) online tools are used to investigate the ADMET features, Lipinski Rule and drug likeness (Mvondo et al., 2021).

### 3.3 Target structure selection and preparation

The 3D crystal protein-structures Monkeypox virus (PDB ID 4QWO) and smallpox virus (PDB 3IGC) were downloaded from the RCSB PDB database (https://www.rcsb.org/). To design any drug, must be needed a receptor. So, the these mentioned target receptor were taken since they are responsible for pathogenic effects. So, if this target protein is possible to inhibited, virus cannot replicate within the host cell and as a result, they cannot produce pathogenicity. Besides, to bind a drug in a specific side, like as lock and key model, must be cleaned or fresh target receptor, excess molecules such as water and other unwanted substances may interfere to bind specific site. So, the water and other unwanted substances are cleaned before docking. By utilizing the Discovery Studio v16.1.0.15350, and Pymol version 2021 program, the 3D crystal structures of Monkeypox and the small virus were cleaned up in preparation for molecular docking. This included the removal of all ligands, non-protein components, and molecules of water (Mahmud et al., 2021). Before molecular docking, the energy of the targeted protein was minimize using swisspdbviwer (Rangisetty et al., 2023).

**Figure 6:**
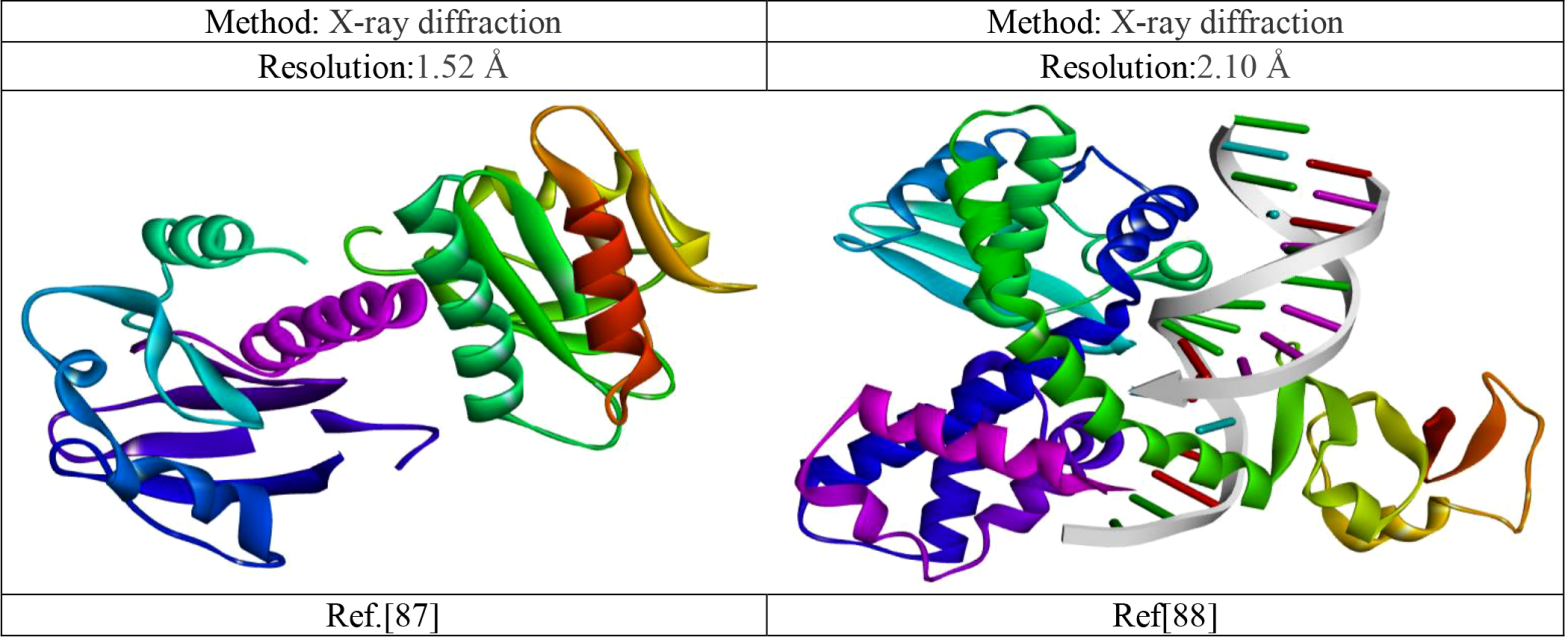
Experimental three-dimensional protein structures information (Minasov et al., 2022; Perry et al., 2010).

The details of Monkeypox and the small virus are listed in **Figure 6**.

### 3.4 Molecular docking analysis

The docking studies modeling was executed to investigate the molecular interaction strategy and acquire documentation on the binding capacity and ligand effectiveness to inhibit the targeted protein. In the current study, the molecular docking analysis was evaluated with the cooperation of the PyRx AutoDock tools application, version 4.2 (Akash et al., 2022a; Rizvi et al., 2013). The automatic maximized function was used to compose the 3D grid for ligand-receptor interaction during molecular docking. The grid box parameters were generated as center (x = 12.0443, y = 18.445, z = 16.0634), dimension (x= 35.14496, y= 37.645, z=36.966) for monkeypox virus, and center (x = 23.2575, y = -9.6147, z = 31.6074), dimension (x= 93.9023, y= 79.8679, z= 63.0821). When the docking analysis was done, The BIOVIA Discovery Studio Visualizer v16.1.0.15350 was applied to generate docked residues, ligand-protein complex structures, and 2D and 3D representations.

### 3.5 Molecular dynamics simulations

YASARA v21.6.17 software was used to run an MD simulation to investigate the relationship between curcumin and 4QWO viral protein. The simulation used the aided model construction with an AMBER 14 force field (Zhang et al., 2020). By combining Cl- and Na^+^ ions, the exchangeable Inter - molecular Potential3 (TIP3P) water model was utilized. Each system operates with the most effective gradient strategy for energy minimization (5000 cycles). The simulations used a periodic boundary condition in which the cell size was always ten times larger than the protein size. Using particle-mesh Ewald (PME) methods, MD simulations and electrostatic interactions were carried out, and also some physiological settings were set at 298 K, 0.9% NaCl, and pH 7.4 (Krieger et al., 2006). A Berendsen thermostat was used to control the simulation temperature while keeping the pressure fixed. Finally, 100 ns of MD simulations were run under constant pressure, and subsequent analysis was handled *via* the built-in YASARA MACRO script (Krieger et al., 2002).

## 4 Results and discussion

### 4.1 Chemistry

The molecular structure of curcumin exposes the myriad of functional groups (1, 7-bis [4-hydroxy-3- methoxyphenyl]-1,6-heptadiene-3,5-dione, **Figure 3**). Rings A and B, both of which are aromatic phenol rings, are joined to one another by different pairs of -unsaturated carbonyl groups (Pullakhandam et al., 2009). These carbonyl groups are excellent Michael receivers and can react with glutathione and other nucleophiles. Other essential structural properties of curcumin are the two aryl methoxy groups located in the ortho position, the hydroxyl component, and the connected -diketone subunits. So, when different substitutes are added, generating various analogs, they act as potential drug candidates and provide better pharmacological efficacy (Nabavi et al., 2014). The studied analogs of curcumin are given in **Figure 3**.

### 4.2 Lipinski rule, pharmacokinetics

The Lipinski rule, commonly referred to as the rule of five, states that one of the most crucial aspects of a drug is how similar it is to other substances. Molecular weight, hydrogen bond donors and acceptors, Topological polar surface area, and Consensus (Log P_o/w_) criteria for determining drug similarity characteristics must be followed (Akash, 2022; Kumer et al., 2022a). The molecular weight should be between 150 and 500 g/mol, the number of hydrogen bond donors should be five or fewer, and the number of hydrogen bond acceptors ought to be ten or fewer. The value of TPSA must fall within the limit of 20 and 130, whereas the acceptable range for Log P_o/w_ must not be higher than 6.00 (Chen et al., 2020).

In our studied compounds, the molecular weight 294.34 – 492.49 Dalton, but two compounds (02 and 27) showed 502.51 Dalton as molecular weight, hydrogen bond donors (03-03) and acceptors (03-08), Topological polar surface area (35.53 Å²-122.16 Å²), and Log P_o/w_ (2.83 – 5.19), which all falls into standard ranges. Lipinski was used to predict the drug likeness accurately, and all the chosen compounds accept the Lipinski rule. So, it is noticed that they might be used as drugs. The *in-silico* prediction of druglike qualities has been compiled in **Table 2**, and it was accomplished with the help of the web program SwissADME.

**Table 2:** Predicted data of Lipinski rule, pharmacokinetics.

Ligand No	Molecular Weight(g/mol)	Hydrogen bond acceptor	Hydrogen bond donor	Topological polar surface area (Å²)	Consensus Log *P*_o/w_	Lipinski rule	
						Result	violation
01	368.38	06	02	93.06	3.03	Yes	00
02	502.51	08	03	122.52	4.16	Yes	01
04	338.35	05	02	83.83	3.00	Yes	00
05	308.33	04	02	74.60	2.83	Yes	00
06	294.34	03	00	35.53	3.86	Yes	00
10	338.35	05	03	86.99	3.18	Yes	00
24	487.50	07	03	122.16	3.61	Yes	00
27	502.51	08	03	122.52	4.16	Yes	01
31	492.47	08	02	93.06	5.05	Yes	00
36	472.49	07	03	113.29	4.05	Yes	00
42	492.47	08	02	93.06	5.19	Yes	00
47	492.47	08	02	93.06	4.99	Yes	00

### 4.3 Molecular docking analysis against monkeypox and smallpox virus

Molecular docking investigation is the key, innovative drug development strategy in the computational chemistry (structure-based drug design), which sorts residual interactions between targeting ligands and a protein’s receptor active site (Ferreira et al., 2015). The docking value is calculated to determine the degree to which ligand molecules bind with the active region of the specific receptor. The greater the negative value of the binding energy, the more strongly preferred the orientation will be, and the more persistent the structure of the ligand-receptor complex is formed (Cerqueira et al., 2015). To become a potential drug candidate, the docking score might be greater than -6.0 kcal/mol (Kumer et al., 2022a; Kumer et al., 2022c). So, the molecular docking was performed against Monkeypox Virus (PDB ID 4QWO) targeted protein initially. When, the binding affinity was achieved outstanding result against Monkeypox Virus (PDB ID 4QWO), then the Smallpox virus (PDB 3IGC) were also taken to determine how much affinity present. In these current studies, the binding affinities are generated from -7.7 kcal/mol to -8.9 kcal/mol against Monkeypox virus (PDB ID 4QWO) and -7.3 kcal/mol to -8.8 kcal/mol against smallpox virus (PDB ID 3IGC). The standard Acyclovir displayed -6.4 kcal/mol and -6.5 kcal/mol. In both cases, the binding energy is more remarkable than -6.00 kcal/mol, much better than standard Acyclovir. Among all derivatives, the best bonding affinity was reported -8.9 kcal/mol against Monkeypox Virus (PDB ID 4QWO) in ligand 24, and -8.8 kcal/mol against Smallpox virus (PDB 3IGC) in ligand 42. So, these reported natural curcumin derivatives should be effective against monkeypox and smallpox viruses.

Noted that fifteen curcumin was taken from PubChem database (Shown in **Supplementary Table 1**), and selected best twelve compounds based on their binding affinities (**Table 3**).

**Table 3:** Summary of binding affinities against Monkeypox, and Smallpox virus.

Drug No	PubChem CID	Monkeypox Virus (PDB ID 4QWO)	Smallpox virus (PDB 3IGC)
		Binding Affinity(kcal/mol)	Binding Affinity(kcal/mol)
01	969516	-7.7	-7.3
02	44195235	-8.2	-8.1
04	146723	-8.1	-6.8
05	147439	-8.2	-7.7
06	830608	-8.1	-7.8
10	5324476	-8.1	-7.3
24	122515213	-8.9	-8.7
27	44195235	-8.2	-8.0
31	162394524	-8.5	-8.2
36	44452370	-8.2	-7.6
42	132993165	-8.8	-8.8
47	54597187	-8.4	-8.7
**Standard (Acyclovir)**	135398513	-6.4	-5.5

### 4.4 Protein-ligand interaction analysis

**Figure 7:**
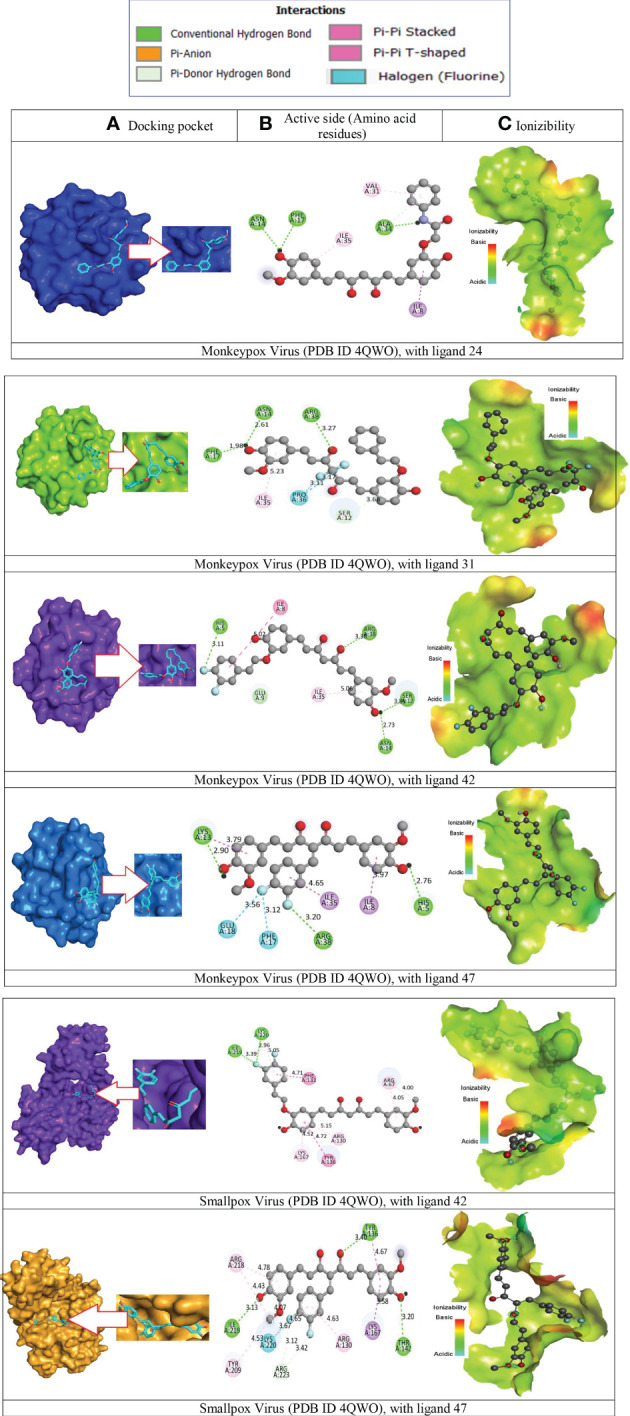
Molecular docking pocket, hydrogen bonding, and binding interaction. **(A)** Docking pocket, **(B)** Active side (Amino acid residues), **(C)** Ionizibility.

The application known as BIOVIA discovery studio visualizer v16.1.0.15350 was used to visualize the interaction and binding between the ligands and the target proteins (Biovia, 2015). When generating protein-ligand complexes, the discovery studio visualizer was used to visualize various non-bonded interactions, such as hydrogen bond interactions and hydrophobic bond interactions with specific distances, as well as active amino acid residues, which are shown in **Figure 7**. The visualization of intermolecular interaction modes to estimate antiviral functions was further investigated so that the mechanism of interactions between bioactive substances and the enzyme substrates specific to each of those medicines could be evaluated.

It has been determined that the significant sites in the instance of monkeypox and smallpox virus constituted the active site pocket are situated at ASN A-14, PHE A-17, ALA A – 34, ILE A – 35, VAL A – 31, ARG A -38, PRO A – 36, LYS A-13, GLU A – 18, HIS A-15, TYR A-136. Besides, the two-dimensional visualizations of the most effective active molecules, as determined by the number of hydrogen bonds formed with the significant amino acids of the Monkeypox and smallpox active sites, are shown in **Figure 7A–C**, correspondingly.

These findings imply that all these ligands might be explored as a viable therapeutic strategy against Monkeypox and smallpox viruses by decreasing viral multiplication and expression. Therefore, the binding of ligands in the active site and their durability are essential factors in determining the possible treatment of Monkeypox and Smallpox viruses. Consequently, they have the capability of monkeypox and smallpox viral agonists since they may potentially attach to the pocket generated by the protein residues of the virus and disrupt the virus’s functionality. The bonding residue of active amino acid for best two complexes are given in **Supplementary Table 1**.

### 4.5 Chemical descriptors calculation

The highest occupied molecular orbitals (HOMOs) and the lowest unoccupied molecular orbitals (LUMO) have a significant role in biochemical reactions. The kinetic resilience, electrochemical stability, chemical hardness, and softness of a drug molecule are all correlated to the HOMO-LUMO energy gap exists between them. The density functional theory was adopted to evaluate the chemical potential, electronegativity, hardness, and softness of our biomolecules (**Table 4**). The HOMO and LUMO frequencies are used to derive these reactivity parameters. The HOMO and LUMO frequencies are used to derive these reactivity parameters. **Table 4** expresses the chemical reactivities of the 12 different curcumin derivatives available in nature.

**Table 4:** Data of chemical descriptors calculation.

S/N	I=- HOMO	A=-LUMO	Energy Gap E(gap) =I-A (eV)	Chemical potential $\left(\mu \right)=\frac{I+A}{2}$	Hardness $\left(\eta \right)=\frac{I-A}{2}$	Softness $\;\;\left(\text{σ}\right)=\frac{1}{\eta }$
01	-9.8934	-0.9844	8.909	-5.4389	4.4545	0.2245
02	-9.886	-1.0205	8.8655	-5.45325	4.43275	0.2256
04	-9.7678	-0.9827	8.7851	-5.37525	4.39255	0.2277
05	-9.7719	-0.9936	8.7783	-5.38275	4.38915	0.2278
06	-8.8939	-0.9682	7.9257	-4.93105	3.96285	0.2523
10	-9.8924	-0.9944	8.898	-5.4434	4.449	0.2248
24	-8.8996	-0.9791	7.9205	-4.93935	3.96025	0.2525
27	-9.896	-0.9952	8.9008	-5.4456	4.4504	0.2247
31	-9.9115	-1.1298	8.7817	-5.52065	4.39085	0.2277
36	-9.0900	-0.9885	8.1015	-5.03925	4.05075	0.2469
42	-9.9041	-1.1225	8.7816	-5.5133	4.3908	0.2277
47	-9.9055	-1.2395	8.666	-5.5725	4.333	0.2308

A larger energy gap suggests significant kinetic stability but low electrochemical stability, while a relatively low energy difference and enhanced softness imply that the molecules have a greater level of polarity and chemical conductivity. Besides, the chemical reactivity of molecules is reduced when the HOMO-LUMO energy gap is large, indicating a lower electronic transition of the electron, and is increased when the gap is small, representing a higher atomic system and higher electrochemical stability, both of which are closely relevant to bind with a targeted protein receptor. The energy gap for organic or aromatic chemicals are typically between 7.00 eV and 9.00 eV, which allows them to attach to any protein efficiency (Kobir et al., 2022). Based on **Table 4**, it is reported that all chemicals maintain the energy gap within the acceptable number (7.00 eV to 9.00 eV). Furthermore, the chemical potential (l), hardness (g), and softness (r), as well as the electronegativity coefficients, might have been utilized to assess therapeutic efficacy (Hoque et al., 2020; Kobir et al., 2022). Usually, the hardness of the material is higher than its softness, and the two properties are inversely proportional to one another. A lower softness score indicates that the components have an outstanding level of dissolving capacity (Kawsar et al., 2022). It is anticipated that the hardness is approximately 3.5 to 4.5, whereas the softness is within 0.24 for all curcumin derivatives. All of the chemical potentials were discovered to be negative, evidence that any form of chemical species or compounds may maintain a satisfactory level of chemical stability and durability. It is clear from looking at **Table 4** that the potential chemical levels of all agonists range from -4.93105 to -5.5725 (Kobir et al., 2022).

### 4.6 Frontier molecular orbitals (HOMO and LUMO)

**Figure 8:**
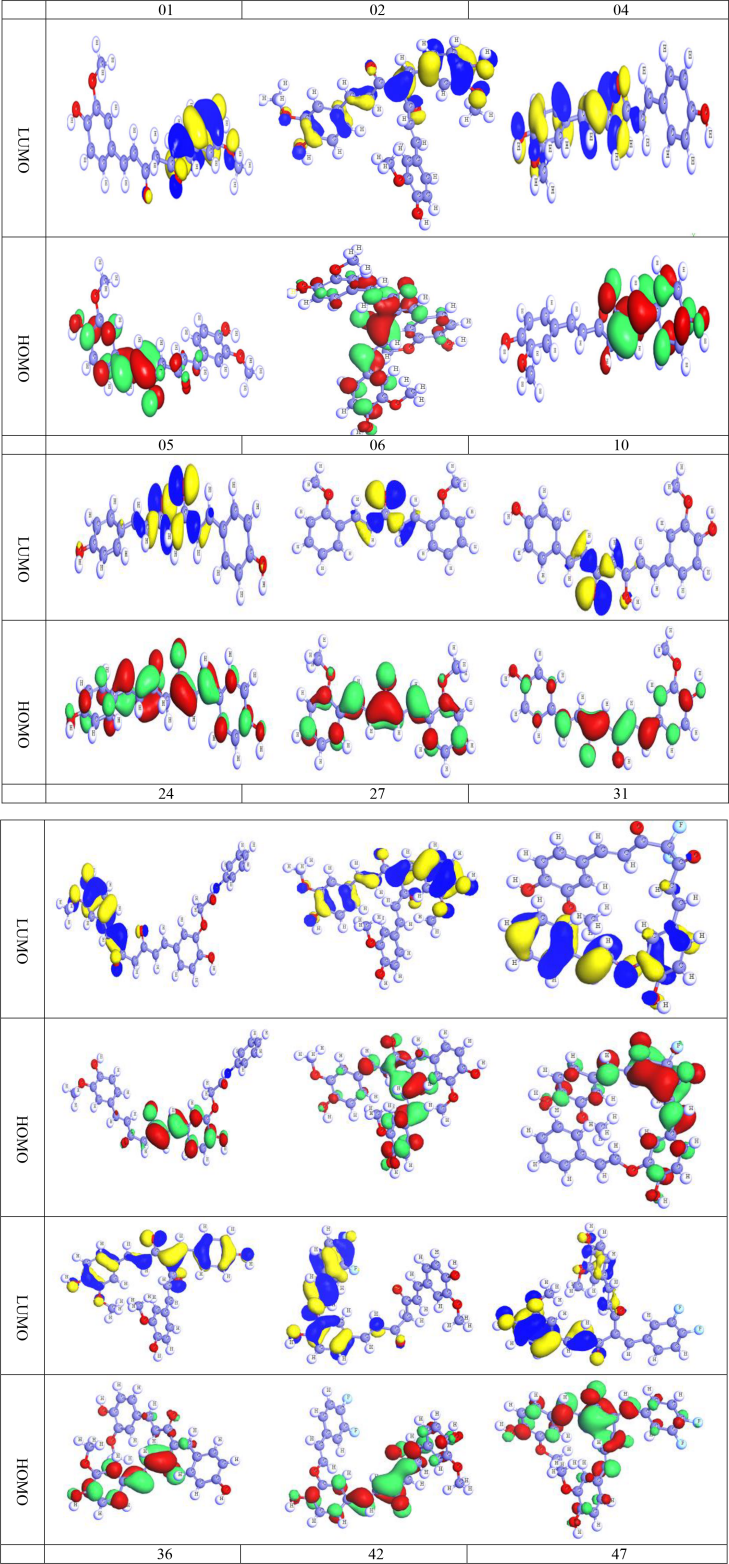
HOMO and LUMO diagram of curcumin and its derivatives.

The HOMO and LUMO are remarkable promoters that can manage the physiochemical properties of any chemical molecule, hence deciding and regulating all of the chemical properties of that substance. From this perspective, it predicts the chemical characteristics of the subsequent curcumin analogs. As before, oxygen atoms in these compounds make it difficult to determine whether the aromatic chain or the heterocyclic ring dominates the chemical characteristics without resorting to the HOMO and LUMO diagrams (Kobir et al., 2022). The discrepancy between HOMO and LUMO is negligible, as seen in **Figure 8**. (The deep blue and yellow of LUMO are the positive and negative ends of the orbital node, and the green and red color of HOMO are the positive and negative ends of the orbital node, similarly). It is remembered that protein or targeted biomolecules are attached in the LUMO region (Kumer et al., 2019; Kumer et al., 2022c).

### 4.7 Molecular dynamics simulations

**Figure 9:**
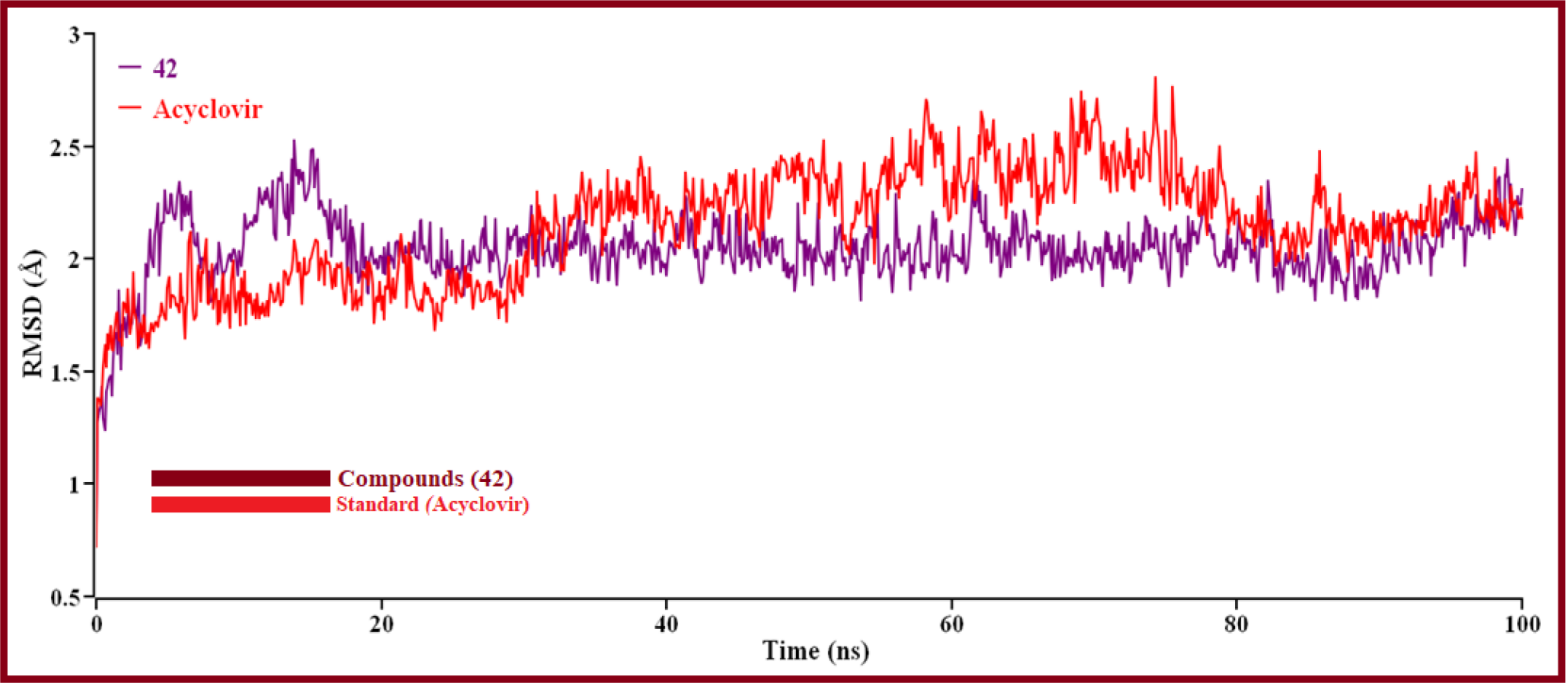
The RMSD of Cα atoms over time for proteins and ligands. Here, red and violet lines denote compound 42 and Acyclovir complexes, respectively.

The hit’s best pose from the virtual screening was chosen compounds (42 and standard Acyclovir), and the YASARA structure’s scenario mode was then set up using the default option (Prasasty and Istyastono, 2020). Here, we ran 100ns of MD simulations using the reference derivatives (Acyclovir). Protein and ligand RMSDs of the Cα atoms were determined and shown in (**Figure 9**) in a time-dependent manner.

**Figure 10:**
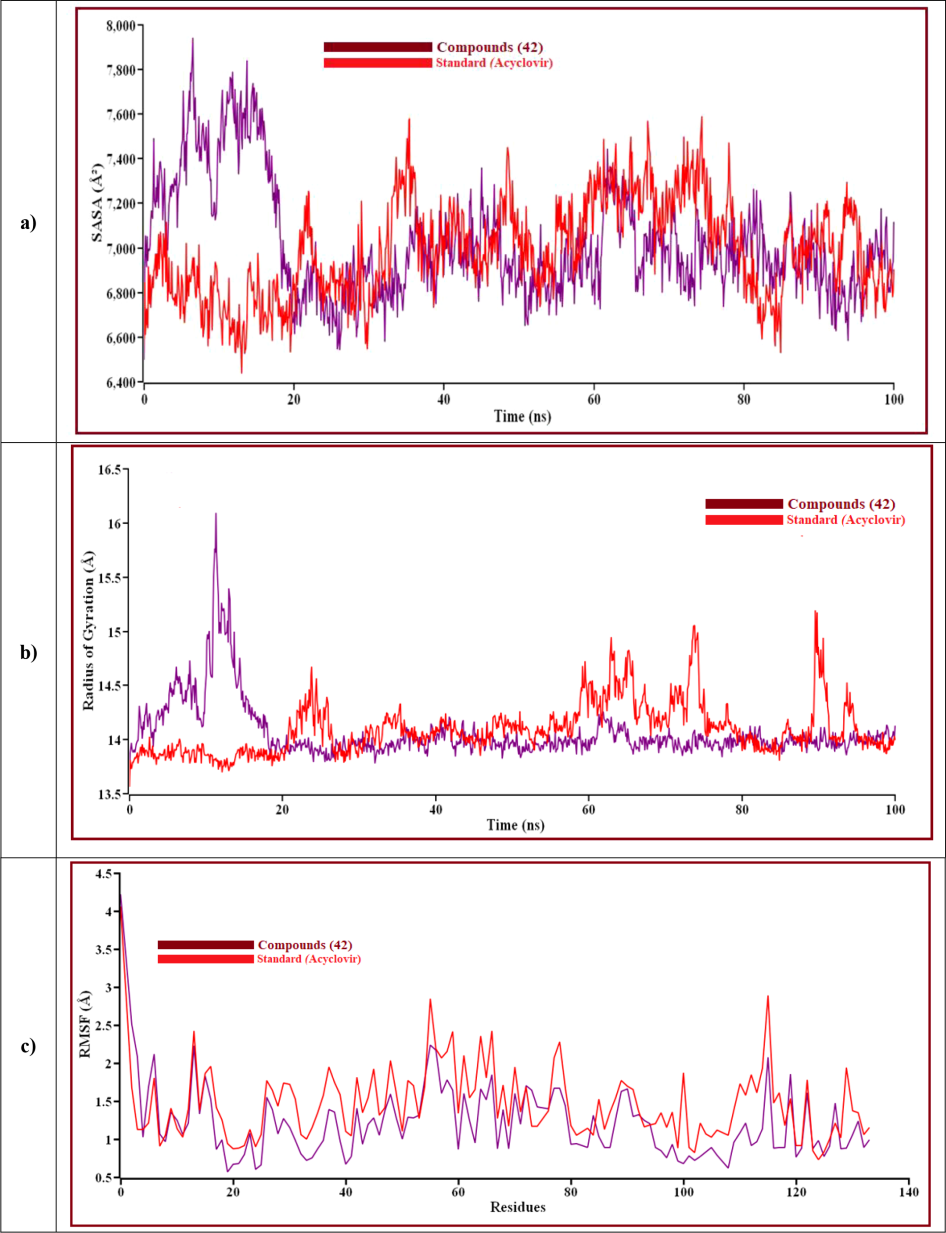
The structural behavior change of protein by means of **(A)** solvent accessible surface area (SASA), **(B)** radius of gyration, and **(C)** root means square fluctuations (RMSF) analysis. Here, the red line indicates Acyclovir, and the violet line indicates compound 42 complexes, respectively.

The result is shown how proteins behave during an MD simulation; as seen in the plot, compound 42 has shown high fluctuation in the primary stage, but after near 74.3ns and 75.5ns, Acyclovir is given the highest range of pick in RMSD, so ligand 42 is a perfectly stable compound. For better results on how ligands 42 and Acyclovir influence the binding mode with Monkeypox protein (PDB ID 4QWO). The two complexes’ structural changes were evaluated by means of root mean square fluctuation (RMSF), the radius of gyration, and the solvent-accessible surface area of the protein-ligand complex (**Figure 10**).

**Figure 11:**
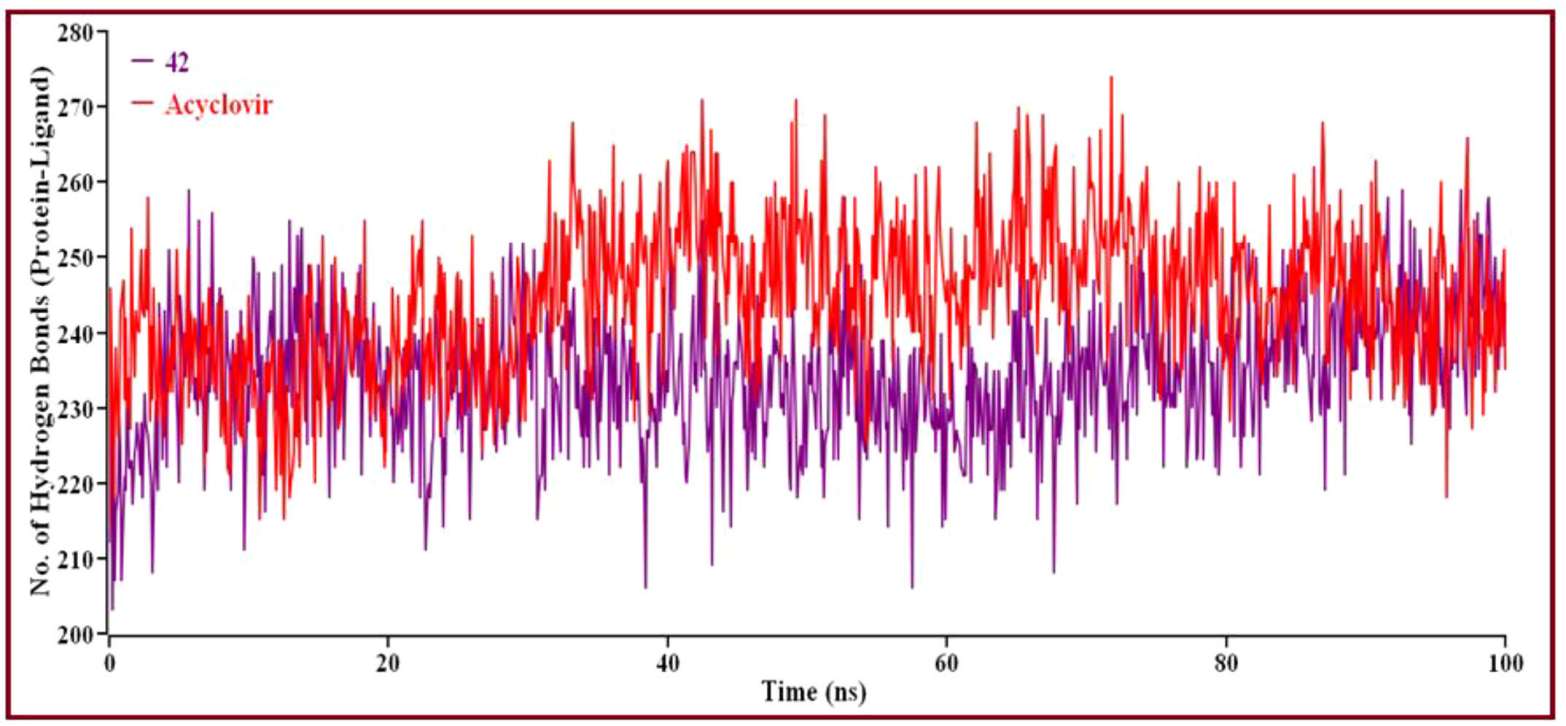
The number of hydrogen bonds created overall between protein-ligand complexes *via* MD simulations. Here, the red line indicates Acyclovir complexes, and violet lines indicate compound 42 complexes, respectively.

Solvent can ruin a pocket if it penetrates the binding site. Protein-ligand interactions must be tightly regulated. **Figure 10A** represents compound 42 showed a high SASA value after close 7ns of simulation, it may not reduce the protein expansion. **Figure 10B** demonstrates the radius of gyration value, compound 42 showed more excellent value than Acyclovir, denoting loose packing of protein structure, RMSF value, which depicts the flexibility of the entire residue in the protein, is shown in **Figure 10C**. High fluctuations were reached in some positions, including (13, 55, 59, 66, and 115 residues), which produced positive results. In **Figure 11**, the hydrogen bond interaction between the protein and the ligand is finally depicted.

Intermolecular hydrogen bonding shows that compound 42 and Acyclovir yield hydrogen bonds with the catalytic domain residue in proteins. Protein folding and de-folding nature depend on intermolecular hydrogen bonds. Acyclovir showed maximum hydrogen bond contact rather than 42 compounds. Eventually, all analyses from the MD simulations suggest that compound 42 is stable and performed a little conformational change of the proteins.

### 4.8 ADME, and aquatic and non-aquatic toxicity

The absorption, distribution, metabolism, excretion, and toxicity of chemicals, abbreviated as ADMET, all contribute a major function significantly to the discovery and development of new drugs. Compounds are absorbed in the human small intestine, move from one tissue to another, undergo chemical biotransformation in the body, and are eliminated from the body *via* excretion; and the level of toxicity of a compound is determined by its toxicity. By using ADMET metrics, we were capable of confirming that the most promising active components against monkeypox and smallpox virus had the potential to become marketable medications. The *in silico* prediction of ADMET characteristics has been compiled in **Table 5**, and it was accomplished with the help of the online program pkCSM.

**Table 5:** Summary of in silico ADMET prediction.

S/N	Absorption			Distribution		Metabolism		Excretion		Toxicity		
	Water solubility Log S	Caco-2 Permeability x 10^-6^	Human Intestinal Absorption (%)	VDss (human)	BBB Permeability	CYP450 1A2 Inhibitor	CYP450 2C9 Substrate	Total Clearance (ml/min/kg)	Renal OCT2 substrate	AMES toxicity	Skin Sensitization	Hepatotoxicity
01	-3.952	0.617	88.035	-0.152	No	No	Yes	0.117	No	No	No	No
02	-3.22	0.66	97.702	-0.445	No	No	Yes	0.112	No	No	No	No
04	-3.967	0.955	92.354	-0.112	No	Yes	Yes	0.099	No	Yes	No	Yes
05	-4.48	1.02	90.805	-0.247	No	Yes	Yes	0.041	No	Yes	No	No
06	-5.346	1.64	95.334	0.015	No	Yes	Yes	0.329	No	No	No	No
10	-3.895	0.894	89.952	0.057	No	Yes	Yes	0.179	No	No	No	Yes
24	-3.8	0.5	82.557	-0.355	No	No	Yes	0.069	No	No	No	No
27	-3.22	0.66	97.702	-0.445	No	No	Yes	0.112	No	No	No	No
31	-4.598	0.497	96.984	-0.639	No	No	Yes	0.012	No	No	No	No
36	-3.543	0.535	95.212	-0.587	No	No	Yes	0.08	No	No	No	No
42	-3.428	0.72	98.664	-0.608	No	No	Yes	-0.246	No	No	No	No
47	-3.301	0.754	100	-0.652	No	No	Yes	-0.243	No	No	No	No

If the absorption score is less than 30 percent, it indicates an inadequate, poor absorption rate. The findings above suggest that the human intestine can absorb all the chemicals effectively, most of which absorb up to 90%. Water solubility (Log S) recommendations for slightly and high solubility compounds vary from -4 to -6 and -2 to -4, correspondingly (Rout et al., 2022). Our invented molecules reported that ligands (05, 06, and 31) are in -4 to - 6 ranges, and the rest ligands ranges fall with -4.0, which means ligands (05, 06, and 31) are slightly soluble, and the others ligands (01,02,04,10,24,30,36,42, and 47) are highly soluble in the water system. Moreover, it is believed that the volume of distribution (VDss) is substantial better if the value is more than 0.45, and our studies reported that most of the curcumin derivatives have a lower volume of distribution (VDss) than the standard. It is also noticed that none of the ligands can cross to the BBB. The biotransformation of pharmaceutical drugs inside the body is referred to as drug metabolism, a phrase often used. The metabolism of drugs results in the formation of a number of distinct enzymatic substrates, each of which contains its unique set of physicochemical, pharmacokinetic, and pharmacological characteristics. As a result, it is essential to consider how the medicine will be metabolized and how it will react with other medications. Drug interactions occur due to cytochrome P450 (CYP) inhibition, which is essential in drug metabolism. The substances 04, 05, 06, and 10 were shown to be inhibitory of the enzymes CYP1A2, while all of the agonists were confirmed to effectively substrate with CYP450 2C9. The clearance parameter characterizes the linear connection between the clearance rate and the plasma concentration of medication. A low clearance constant thus suggests enhanced retention of medicines inside the body long time. The cumulative clearance rate of all chemicals demonstrates that the medicine can excrete from the body after an extended period. At the same time, only ligands 42 and 47 have negative scores, which means these two have poor excretion rate constants. Finally, carcinogenicity is applied to characterize the toxicity of AMES; hence, it is essential to ensure that the expected chemicals are not carcinogenic, skin sensitization, or hepatotoxicity. Our projection is predicted that only ligands 04 and 05 may be carcinogenic, and ligands 04 and 10 may be the hepatotoxic effect, while the rest ligands are free from skin sensitization effects. So, the overall finding of ADMET is favorable and suggests them as new drug candidates.

## 5 Conclusions

Fifty different natural curcumin derivatives were used in these computational investigations. In the meantime, twelve compounds were chosen based on having the highest possible docking score. After that, a multitude of computational studies were carried out, including molecular docking, dynamic modeling, ADMET, and DFT.The molecular docking investigation corroborated these findings, showing promise antiviral effectiveness against monkeypox and smallpox virus. The finding docking score is about -7.7 kcal/mol to -8.9 kcal/mol against monkeypox virus (PDB ID 4QWO) and -7.3 kcal/mol to -8.8 kcal/mol against smallpox virus (PDB ID 3IGC). During molecular docking, different types of bond and active amino acid residues were formed like as ASN A-14, PHE A-17, ALA A – 34, ILE A – 35, VAL A – 31, ARG A -38, PRO A – 36, LYS A-13, GLU A – 18, HIS A-15, TYR A-136. After that, DFT calculation, the curcumin derivatives have shown the hardness is approximately 3.5 to 4.5, whereas the softness is within 0.24 for all curcumin derivatives. So, it is suggested that these derivatives may easily decompose or breakdown within physiological system. Then, the molecular dynamic simulations were performed. The MDs significantly and emphatically corroborates this observation over 100 ns, which confirms the binding stability of the docked complexes in the trajectory analysis. It suggests that the protein-ligand complexes maintain the strongest stability inside the biological system. In accordance with this, the pharmacological features of these chemicals suggested that the majority of the developed molecules demonstrated enhanced pharmacokinetic parameters, conserved all drug-likeness rules, and increased pharmacological activities. Based on their pharmacokinetic and biological profiles, reported curcumin derivatives were shown to have the most promising use in treating Monkeypox and smallpox viral infections. So, these mentioned derivatives might be further suggested for experimental animal model.

## Data availability statement

The datasets presented in this study can be found in online repositories. The names of the repository/repositories and accession number(s) can be found in the article/**Supplementary Material**.

## Author contributions

*Conceptualization*, SA, AH, and MR. *methodology*, SA, MH. *validation*, SA, MH. *formal analysis*, SA, MH, and MR. *data curation*, SA, and MR. *writing—original draft preparation*, SA. *writing—review and editing*, MA, NA, MV, KK, RS. *supervision and project administration*, RS. All authors contributed to the article and approved the submitted version.
