# Corrigendum: Anti-viral drug discovery against monkeypox and smallpox infection by natural curcumin derivatives: a computational drug design approach

**DOI:** 10.3389/fcimb.2023.1217334

**Published:** 2023-06-09

**Authors:** Shopnil Akash, Arafat Hossain, Md. Sarowar Hossain, Md. Mominur Rahman, Mohammad Z. Ahmed, Nemat Ali, Martin Valis, Kamil Kuca, Rohit Sharma

**Affiliations:** ^1^ Department of Pharmacy, Faculty of Allied Health Science, Daffodil International University, Dhaka, Bangladesh; ^2^ Department of Biochemistry and Molecular Biology, Bangabandhu Sheikh Mujibur Rahman Science and Technology University, Gopalganj, Bangladesh; ^3^ Department of Pharmacognosy, College of Pharmacy, King Saud University, Riyadh, Saudi Arabia; ^4^ Department of Pharmacology and Toxicology, College of Pharmacy, King Saud University, Riyadh, Saudi Arabia; ^5^ Department of Neurology, Medical Faculty, Charles University and University Hospital in Hradec Králové, Hradec Králové, Czechia; ^6^ Department of Chemistry, Faculty of Science, University of Hradec Králové, Hradec Králové, Czechia; ^7^ Biomedical Research Center, University Hospital Hradec Kralove, Hradec Kralove, Czechia; ^8^ Department of Rasa Shastra and Bhaishajya Kalpana, Faculty of Ayurveda, Institute of Medical Sciences, Banaras Hindu University, Varanasi, Uttar Pradesh, India

**Keywords:** curcumin, monkeypox, smallpox virus, molecular docking, DFT, admet, molecular dynamic simulation

## Text correction 1

In the published article, there was an error. The error in our article is in the abstract section. Before 6.8 kcal/mol, a minus (-) sign will be added and it will be -6.8 kcal/mol. A correction has been made to **Abstract, Result, Three.**

“The mentioned derivatives demonstrated docking scores greater than 6.80 kcal/mol, and the most significant binding affinity was at -8.90 kcal/mol, even though 12 molecules had higher binding scores (-8.00 kcal/mol to -8.9 kcal/mol), and better than the standard medications”.

The corrected sentence appears below:

“The mentioned derivatives demonstrated docking scores greater than -6.80 kcal/mol, and the most significant binding affinity was at -8.90 kcal/mol, even though 12 molecules had higher binding scores (-8.00 kcal/mol to -8.9 kcal/mol), and better than the standard medications”.

The authors apologize for this error and state that this does not change the scientific conclusions of the article in any way.

## Text correction 2

In the published article, there was an error. The 2nd error in our article is in the literature review section. The sub section 2.7 Effects of Curcumin on herpes simplex virus should be Effects of Curcumin on Hepatitis C virus instead of herpes simplex virus.

A correction has been made to Literature review, Effects of Curcumin on herpes simplex virus, 0.

“Effects of curcumin on herpes simplex virus”.

The corrected sentence appears below:

“Effects of Curcumin on Hepatitis C virus instead of herpes simplex virus”.

The authors apologize for this error and state that this does not change the scientific conclusions of the article in any way.

