# Supplementary material for: Anti-viral drug discovery against monkeypox and smallpox infection by natural curcumin derivatives: A Computational drug design approach
Source: Front Cell Infect Microbiol. 2023 Mar 22;13:1157627. doi: 10.3389/fcimb.2023.1157627 (PMC10073709; doi:10.3389/fcimb.2023.1157627)
Supplement: Supplementary file 1 [file Table_1.docx]

| Supplementary Table 1 | | |
| --- | --- | --- |
| Drug No | PubChem CID | Monkeypox Virus (PDB ID 4QWO) |
|  |  | Binding Affinity(kcal/mole) |
|  | 2889 | -7.6 |
|  | 44195235 | -8.2 |
|  | 124072 | -7.7 |
|  | 146723 | -8.1 |
|  | 147439 | -8.2 |
|  | 830608 | -8.1 |
|  | 969516 | -7.7 |
|  | 5281767 | 7.8 |
|  | 5318039 | -7.8 |
|  | 5324476 | -8.1 |
|  | 9952605 | -7.2 |
|  | 11068034 | -7.0 |
|  | 11474949 | -7.7 |
|  | 11947775 | -7.1 |
|  | 16727530 | -7.0 |
|  | 24766776 | -7.5 |
|  | 24884282 | -8.0 |
|  | 25111343 | -6.9 |
|  | 44451939 | -8.1 |
|  | 45276266 | -7.1 |
|  | 45276267 | -6.8 |
|  | 53464495 | -8.0 |
|  | 122515213 | -8.9 |
|  | 123810742 | -7.8 |
|  | 124109563 | -7.6 |
|  | 44195235 | -8.2 |
|  | 129728794 | -7.1 |
|  | 129738071 | -7.6 |
|  | 129826075 | -7.5 |
|  | 162394524 | -8.5 |
|  | 1224938809 | -7.0 |
|  | 87090756 | -8.0 |
|  | 87261197 | -7.8 |
|  | 87401562 | -8.0 |
|  | 44452370 | -8.2 |
|  | 87701378 | -7.6 |
|  | 88107920 | -7.4 |
|  | 88539422 | -7.9 |
|  | 89978543 | -7.7 |
|  | 135440402 | -8.0 |
|  | 132993165 | -8.8 |
|  | 135494223 | -7.5 |
|  | 136036824 | -8.0 |
|  | 136036825 | -8.0 |
|  | 136036827 | -8.0 |
|  | 54597187 | -8.4 |
|  | 137250306 | -7.6 |
|  | 137314529 | -7.1 |
|  | 138374100 | -7.8 |
|  | 141308572 | -7.9 |
